# Clinical Value of Optical Coherence Tomography in Craniopharyngioma

**DOI:** 10.3390/cancers18061030

**Published:** 2026-03-23

**Authors:** Klaudia Rakusiewicz-Krasnodębska, Agnieszka Bogusz-Wójcik, Anna Chmielarz-Czarnocińska, Elżbieta Moszczyńska, Wojciech Hautz

**Affiliations:** 1 Department of Pediatric Ophthalmology, Children’s Memorial Health Institute, 04-730 Warsaw, Poland; 2 Department of Pediatric Endocrinology and Diabetology, Children’s Memorial Health Institute, 04-730 Warsaw, Poland; 3 Department of Ophthalmology, Poznan University of Medical Sciences, 61-848 Poznan, Poland

**Keywords:** craniopharyngioma, optical coherence tomography (OCT), OCT angiography (OCTA), retinal nerve fiber layer (RNFL), ganglion cell complex (GCC), visual outcomes, chiasmal compression, pediatric ophthalmology

## Abstract

Craniopharyngioma (CP) is a rare benign tumor of the sellar and suprasellar region that often compresses the optic pathways, causing significant visual impairment in both children and adults. The early detection and monitoring of optic nerve involvement are essential for preserving visual function. Optical coherence tomography (OCT) and OCT angiography (OCTA) are noninvasive, high-resolution imaging modalities that provide quantitative assessment of retinal nerve fiber layer (RNFL) thickness, ganglion cell complex (GCC), and retinal microvasculature. Thinning of the RNFL and GCC correlates with visual field defects and reduced visual acuity and may also serve as a predictor of postoperative visual recovery. OCTA reveals microvascular alterations that may precede structural damage and, together with other imaging parameters, can be used to estimate the likelihood of visual improvement after neurosurgery. This review summarizes current evidence on the use of OCT and OCTA in CP, highlighting their applications in assessment of optic pathway involvement, preoperative evaluation, postoperative monitoring, and risk stratification. Based on our clinical experience, we propose a table with recommended OCT parameters and follow-up intervals. Importantly, OCT should be interpreted alongside the visual acuity, visual field testing, and fundus examination for comprehensive assessment. Future directions include the standardization of imaging protocols and prospective multicenter studies, and integration of OCTA metrics into predictive models of visual outcomes. OCT and OCTA provide objective, reproducible biomarkers that support individualized patient care and may improve visual prognosis in CP.

## Simple Summary

Craniopharyngioma is a rare benign brain tumor that often develops near the optic nerves and optic chiasm, which can lead to significant vision problems in both children and adults. Early detection of optic nerve damage is important to preserve visual function. Optical coherence tomography (OCT) and OCT angiography (OCTA) are noninvasive imaging techniques that allow detailed evaluation of the retina and its blood vessels. Changes such as thinning of the retinal nerve fiber layer and ganglion cell complex are associated with visual impairment and may help predict visual recovery after neurosurgery. OCTA can also detect microvascular alterations that may appear before structural damage. This review summarizes current knowledge about the role of OCT and OCTA in diagnosis, monitoring, and prognosis of visual outcomes in patients with craniopharyngioma.

## 1. Introduction

Craniopharyngioma (CP) is a rare, benign epithelial tumor arising in the sellar and suprasellar regions, accounting for a small proportion of intracranial neoplasms in both pediatric and adult populations [1,2,3,4]. This tumor shows a bimodal age distribution, with peak incidence in childhood between 5 and 15 years of age and a second peak in adults between 45 and 60 years [1,3]. Although CPs are tumors of low histological malignancy (WHO grade 1), their anatomical proximity to critical structures, including the optic chiasm, hypothalamus, and pituitary gland, results in substantial morbidity. Its slow growth often leads to progressive compression of adjacent neural structures, resulting in a wide spectrum of clinical manifestations [3,4,5,6,7,8]. While the neurological and endocrinological consequences of CP are well documented, its ophthalmic implications are equally significant yet sometimes underrecognized [9,10,11,12].

Compression of the optic pathways can lead to substantial visual impairment [7,13,14,15,16,17,18]. Characteristic ocular manifestations include visual field defects, most commonly bitemporal hemianopia, reduced visual acuity, and optic disk changes such as pallor and atrophy. Importantly, ophthalmic symptoms often precede other clinical signs, including endocrine disturbances, making them a key element in early detection [10,13,19,20]. It is estimated that in approximately 62–84% of cases, ocular symptoms represent the initial manifestation of CP and play a key role in establishing diagnosis [13,20,21,22]. Subtle visual complaints in children, such as difficulty reading, clumsiness, or squinting can be early indicators of optic pathway involvement, highlighting the importance of thorough ophthalmic evaluation in suspected cases. Moreover, visual deficits substantially affect patients’ everyday activities and overall quality of life following treatment for CP [22].

Over the past decade, optical coherence tomography (OCT) has emerged as a noninvasive, high-resolution imaging modality that allows precise evaluation of retinal and optic nerve structures [23,24,25]. OCT provides qualitative and, importantly, quantitative measurements of retinal nerve fiber layer (RNFL) thickness and ganglion cell complex (GCC) integrity, which are key indicators of optic nerve health [26]. Increasing evidence indicates that these parameters have predictive value for postoperative visual recovery, providing clinicians with a tool to stratify risk and guide surgical planning [27,28,29,30,31,32]. Furthermore, advancements in OCT angiography (OCTA) have enabled the visualization of retinal and optic nerve head microvasculature, allowing early detection of perfusion deficits that may precede overt structural or functional damage [33,34,35].

This review aims to summarize the current evidence regarding OCT and OCTA findings in patients with CP and to discuss their application in clinical practice. By consolidating structural and microvascular changes identified with these modalities, we aim to provide guidance for ophthalmic assessment, preoperative evaluation, postoperative monitoring, and prognostication in patients affected by this challenging tumor.

## 2. Methods

This review is a narrative review based on a structured literature search. The literature search was performed in the PubMed and Scopus databases to identify studies evaluating optical coherence tomography (OCT) and visual pathway assessment in patients with CP. The search included articles published in English using combinations of the following keywords: “craniopharyngioma”, “optical coherence tomography”, “retinal nerve fiber layer”, “ganglion cell complex”, and “compressive optic neuropathy”. Original research articles, clinical studies, and relevant review papers focusing on ophthalmological assessment in patients with CP were included. Articles not related to visual pathway assessment, non-human studies, and publications without accessible full text were excluded. The selection of studies was based on their relevance to the clinical application of OCT in the diagnosis, monitoring, and prognostic evaluation of visual function in CP.

## 3. Pathophysiology

CPs arise from epithelial remnants of Rathke’s pouch and are typically located in the sellar and suprasellar regions, in proximity to the optic nerves and optic chiasm [1,36,37]. Although histologically benign, their characteristic growth pattern frequently places them directly adjacent to, or even encasing, the optic chiasm and surrounding neurovascular structures, leading to visual impairment as a common clinical manifestation. As the tumor enlarges, it progressively exerts a mass effect on the optic pathways. In the early stages, visual dysfunction is primarily related to mechanical compression of the optic nerves and chiasm, leading to functional impairment that may remain at least partially reversible [7,15,27]. Chronic compression leads to irreversible injury, including disruption of axoplasmic flow within retinal ganglion cell (RGC) axons, mitochondrial dysfunction, and activation of apoptotic pathways, resulting in retrograde and anterograde axonal degeneration and optic nerve atrophy [31,38,39,40]. Mechanical compression produces sectoral thinning of the peripapillary RNFL and localized GCC loss corresponding to affected fiber topography.

**Figure 1:**
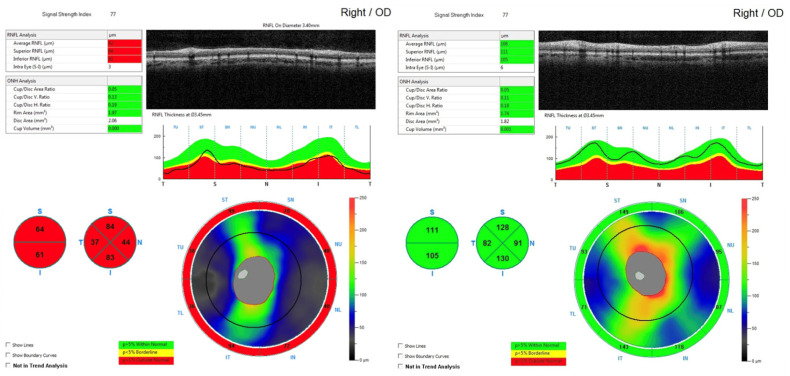
OCT scan of the retinal nerve fiber layer (RNFL) surrounding the optic nerve in the eye. Diffuse damage across all sectors is observed in a patient with optic chiasm compression caused by craniopharyngioma. Partially reproduced from [45].

**Figure 2:**
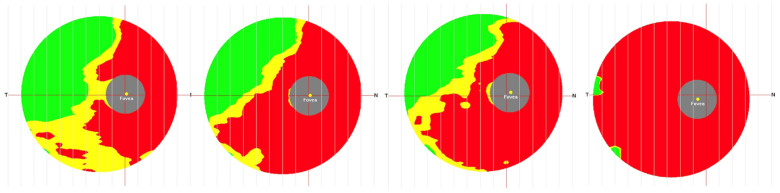
Serial ganglion cell complex (GCC) scans in a patient with optic chiasm compression, demonstrating progression of GCC damage.

In addition to mechanical compression, vascular compromise plays an important role in the pathophysiology of visual loss in CP. Compression of small vessels supplying the optic nerves and chiasm may impair microperfusion, contributing to ischemia and metabolic stress, which further exacerbate axonal damage and neuronal loss. These combined mechanisms, mechanical, ischemic, and metabolic, are reflected structurally by thinning of the peripapillary RNFL [Figure 1] and loss of the GCC [Figure 2], as demonstrated by OCT [15,17,41,42]. Sectoral RNFL and GCC loss often correlates with visual field deficits, linking structural and functional impact. Importantly, despite severe initial injury, portions of the visual pathways may retain latent functional capacity, providing a potential substrate for visual recovery following timely surgical decompression [43,44].

## 4. Ocular Manifestation

Ophthalmic manifestations are among the most frequent and clinically significant presentations of CP, particularly in pediatric patients [1,7,15,16,17,19,20,21]. Up to 62–84% of patients present with ophthalmological symptoms at the time of diagnosis [19,46]. However, deterioration in vision is reported in 3.7% of patients after surgical treatment, and in 8.6% of patients undergoing fractionated radiotherapy or stereotactic radiosurgery separately [12,47]. Visual field defects are among the most characteristic findings, with bitemporal hemianopia being the predominant pattern observed, reflecting involvement of the central optic pathways [7]. According to large meta-analyses, visual field defects are present at diagnosis in approximately 38.3% of children with CP, and bitemporal hemianopia remains a hallmark clinical sign [21].

Reduced visual acuity is among the most frequently reported ophthalmic manifestations at the time of diagnosis. At presentation, decreased visual acuity is observed in 41.3% of patients and may occur asymmetrically, reflecting heterogeneous involvement of the visual pathways [21]. Notably, unilateral or bilateral blindness is reported in approximately 13.8% of pediatric patients, underscoring the potential severity of visual impairment at initial evaluation. Visual acuity may improve following surgical treatment and decompression of the optic chiasm, with postoperative improvement reported in 47% of patients [44,48,49,50]. Nevertheless, visual impairment remains one of the most devastating sequelae of CP, ranging from mild reductions in visual acuity to profound visual loss and blindness, with a substantial impact on quality of life [20,51,52,53].

Fundoscopic abnormalities are also frequently observed during ophthalmological examination. According to available data, abnormal findings on fundus examination are detected in approximately 32.5% of patients and include optic disk swelling in earlier stages, as well as optic disk pallor or established optic nerve atrophy in more chronic cases [54]. These structural changes are indicative of longstanding visual pathway involvement and are often associated with poorer visual prognosis.

Ocular motility disturbances and orthoptic abnormalities constitute another important component of the ophthalmic spectrum, particularly in children. Strabismus, impaired ocular alignment, and disturbances of eye movements are reported in approximately 32.5% of pediatric patients and may be accompanied by abnormal saccadic function. These findings further contribute to functional visual impairment and may complicate both diagnosis and long-term management. Other ophthalmic symptoms that occur less frequently in patients with CP include: diplopia, nystagmus, pupillary abnormalities, and photophobia [8,20,55].

The cumulative burden of visual field loss, reduced visual acuity, optic nerve damage, and ocular motility disturbances has a profound impact on patients’ daily functioning, educational performance, and overall quality of life, persisting even after surgical treatment in a substantial proportion of cases [8,12,20,22].

## 5. OCT in Craniopharyngioma

OCT, a noninvasive, high-resolution imaging modality that enables in vivo cross-sectional visualization of retinal microstructure in patients with CP, provides an objective assessment of the anterior visual pathway by quantifying structural changes secondary to optic nerve and chiasmal involvement [24,25,26]. Standard OCT allows measurement of peripapillary RNFL thickness and macular GCC, which reflect axonal and neuronal integrity, respectively [56,57,58]. OCTA extends structural imaging by enabling dye-free evaluation of retinal and peripapillary microvasculature, including vessel density and perfusion parameters [33,34,59,60]. Both OCT and OCTA are highly reproducible and objective techniques, characterized by low operator dependency and minimal susceptibility to measurement error [61,62,63]. Importantly, these modalities allow the assessment of focal structural damage at a single time point as well as longitudinal monitoring of dynamic changes over time, making them valuable tools for diagnosis, prognostication, and follow-up in patients with CP.

## 6. Assessment of Retinal Nerve Fiber Layer in Patients with CP

It is well established that in patients with optic chiasm compression, mechanical pressure leads to retinal ganglion cell loss, which is manifested as thinning of the peripapillary RNFL [18,32,54,64,65,66,67]. Assessment of RNFL thickness using OCT represents one of the most robust and clinically relevant methods for evaluating optic pathway damage in patients with CP. Patients with CP consistently demonstrate significantly reduced RNFL thickness compared with healthy controls [31,32,64,68]. RNFL thickness reflects the integrity of retinal ganglion cell axons and serves as a structural surrogate marker of optic nerve and chiasmal involvement [69,70,71]. Importantly, characteristic sectoral patterns of RNFL thinning associated with chiasmal compression, particularly involving nasal fibers, provide diagnostic value and may help differentiate chiasmal pathology from other optic neuropathies [54,64,72]. Sectoral RNFL analysis enables detection of these patterns even in cases with subtle or asymmetric functional deficits [66,72,73,74]. As expected based on anatomy, parameters in the nasal hemiretina demonstrated a greater ability to detect damage in eyes with chiasmal compression than those in the temporal hemiretina [66,73,74]. Moon et al. [72] reported damage predominantly in the nasal and temporal sectors in patients with neuropathy caused by chiasmal compression, whereas more pronounced damage in the superior and inferior sectors was observed in patients with glaucoma-related neuropathy. In the same study, they reported a significantly higher RNFL score in patients with optic chiasm compression than in patients with glaucoma and those suspected of having glaucoma [72]. OCT, owing to its high repeatability, objectivity, and minimal dependence on patient cooperation, allows reliable detection of RNFL changes suggestive of optic chiasm compression, thereby enhancing the diagnostic workup and supporting accurate diagnosis, particularly in uncooperative patients [13,75,76]. RNFL very often correlates with visual acuity, but not invariably, which is particularly useful in children and in patients in whom assessment of visual acuity or visual fields is not feasible [75]. In the study by Rakusiewicz-Krasnodębska et al. [64], the degree of RNFL damage correlated with tumor volume, maximum tumor diameter, calcification, ventriculoperitoneal shunt placement, surgical technique, extent of resection, presence of Rosenthal fibers, and reoperation due to tumor progression or recurrence. Similarly, Bogusz-Wójcik et al. [45] reported correlations between RNFL thinning and inappropriate secretion of antidiuretic hormone, arginine vasopressin deficiency, memory disorders, and hyperphagia after surgery, all of which were associated with RNFL damage. The RNFL assessment demonstrated a sensitivity of 95.83% and a specificity of 27.59% for the diagnosis of chiasmal compression [72]. Furthermore, OCT enables long-term postoperative monitoring of disease progression and visual outcomes in both adult and pediatric patients with CP [14,31,75]. Furthermore, it is emphasized that preoperative RNFL assessment may also have prognostic value for final visual outcomes, including postoperative visual acuity and visual field recovery, with preserved RNFL being associated with a greater likelihood of visual improvement after surgical decompression [14,18,30,32,40,66,68]. For an overview of the evidence, Table 1 presents clinical studies evaluating peripapillary RNFL thickness with OCT in patients with CP.

## 7. Assessment of Ganglion Cell Complex (GCC) in Patients with CP

Assessment of the GCC using OCT provides a sensitive structural measure of retinal ganglion cell bodies and their dendritic connections within the inner plexiform layer [79]. In patients with CP, GCC analysis enables detection of early neuronal damage resulting from optic chiasm compression, often preceding or exceeding changes observed in peripapillary RNFL thickness [77,80]. Several studies have demonstrated that CP, particularly when involving the optic pathways, leads to significant structural damage of the visual system, most commonly manifesting as GCC thinning in both pediatric and adult patients [14,45,73]. The degree and topographic pattern of GCC loss frequently correlates with functional visual impairment, including visual field defects and reduced visual acuity [13,75]. Due to the characteristic anatomy of the optic chiasm and its preferential involvement of the central fibers, macular parameters in the nasal hemiretina demonstrate a greater ability to detect damage in eyes with chiasmal compression than those in the temporal hemiretina [73]. Chronic compression of the optic pathways may result in retrograde axonal degeneration, leading to progressive loss of retinal ganglion cells and subsequent irreversible visual impairment. In this context, several authors have emphasized the superior diagnostic value of GCC analysis compared with the more commonly used peripapillary RNFL, citing higher sensitivity and specificity for detecting optic pathway damage. Moreover, Yoo et al. [81] reported that GCC thickness measured by OCT was a better predictor of postoperative visual outcomes in parasellar tumors than RNFL thickness, supporting its role as a robust prognostic marker of visual recovery following chiasmal decompression [77,80]. Similarly, Moon et al. [40] demonstrated that GCC thickness correlated more strongly with visual field defects than mean RNFL thickness in patients with sellar tumors, concluding that selective assessment of the GCC may represent a more reliable parameter for evaluating chiasmal compression related damage. Importantly, preserved preoperative GCC thickness has been associated with better postoperative visual outcomes, and this correlates with final visual acuity and visual field, highlighting its potential value in surgical decision making [75]. From a practical perspective, GCC assessment at the macular level remains feasible even in patients with low visual acuity and poor fixation, whereas RNFL evaluation, which requires stable peripheral fixation, is often technically challenging. In addition, RNFL measurements may be confounded by optic disk edema in patients with CP, potentially resulting in falsely elevated values and reduced diagnostic accuracy [82]. Owing to its high reproducibility, objectivity, and lower susceptibility to segmentation artifacts GCC analysis complements RNFL by providing reliable information on macular neuronal integrity and represents a valuable tool for the diagnosis, monitoring, and assessment of disease progression or stability in patients with CP. Table 2 summarizes clinical studies evaluating the GCC using OCT in patients with CP and chiasmal compression.

## 8. OCT Angiography (OCTA) in CP

OCTA enables noninvasive, dye-free visualization and quantitative assessment of the retinal and optic nerve head microvasculature, providing complementary information to structural OCT in patients with CP [33,35,61,63,83]. OCTA allows evaluation of circumpapillary and macular vessel density, foveal avascular zone (FAZ) metrics, and capillary perfusion within superficial and deep retinal plexuses [84,85,86]. Studies in patients with chiasmal compression, including small CP cohorts, have demonstrated significant reductions in peripapillary and parafoveal vessel density, which correlate with retinal neural loss, visual field defects, and decreased visual acuity [14,34,41,42,60,87]. In adults, reduced peripapillary and macular perfusion has been shown to correlate with temporal hemianopia, visual field loss, and the severity of optic nerve compression, supporting the concept of compression-related retinal hypoperfusion as a marker of axonal injury [14,41,60,88,89]. Importantly, OCTA-derived microvascular alterations, such as reduced capillary density and impaired choroidal perfusion, are presumed to have prognostic value, with preserved vascular parameters potentially associated with better postoperative visual outcomes [41,60,89]. Emerging evidence suggests that microvascular changes detected by OCTA may precede overt structural thinning on conventional OCT, indicating a potential role in early detection of optic pathway compromise, risk stratification, and longitudinal monitoring of disease progression and treatment response in patients with CP, although these findings require confirmation in larger, CP-specific cohorts. Furthermore, longitudinal OCTA studies demonstrate partial recovery of retinal and peripapillary perfusion following surgical decompression, which parallels improvements in RNFL/GCC thickness and visual function [42,88], supporting the role of OCTA in postoperative monitoring. Collectively, these findings support OCTA as a noninvasive modality that complements structural OCT by capturing the microvascular component of chiasmal compression–related optic neuropathy and by providing clinically relevant prognostic and longitudinal information in patients with CP. OCTA enables noninvasive evaluation of retinal and optic nerve microvasculature. The relevant studies are summarized in Table 3.

## 9. OCT Clinical Applications

### 9.1. The Role of OCT in the Diagnosis of CP

A well-known clinical feature among specialists involved in the diagnosis of CP, including endocrinologists, neurologists, neurosurgeons, and ophthalmologists, is bitemporal hemianopia resulting from tumor-related compression of the optic chiasm. This visual field defect is associated with measurable thinning of the RNFL and the GCC on ophthalmic imaging [8,22,49]. OCT enables quantitative and objective assessment of both RNFL and macular GCC thickness, providing valuable metrics for evaluating the severity of optic pathway compression demonstrated significant RNFL thinning in both pediatric and adult patients with primary CP compared with healthy controls, reflecting early axonal loss secondary to chiasmal compression [13,31,73,77]. Similarly, Mediero et al. [13] reported that GCC thinning in pediatric patients with CP correlated with visual field defects and reduced visual acuity, underscoring the sensitivity of GCC analysis in detecting subclinical neuronal damage. Macular SD-OCT analysis further refines detection by identifying topographic patterns of ganglion cell loss, particularly in regions affected by naso-temporal fiber overlap [73]. In clinical practice, the presence of RNFL and GCC damage on OCT, especially when accompanied by neurological or visual symptoms, should prompt further diagnostic evaluation with brain MRI, which remains the gold standard for identifying CP [1,8,36]. It is important to note that while OCT can indicate the presence of compressive optic neuropathy, it cannot differentiate CP from other parasellar lesions. Many of the studies cited in this review include mixed sellar tumors, limiting the generalizability of their conclusions to CP. Overall, OCT represents a noninvasive, reproducible, and sensitive tool for detecting optic pathway involvement in CP and supports individualized clinical decision-making.

### 9.2. Predictive Value and Correlation of OCT Parameters with Visual Function in Craniopharyngioma

Beyond its invaluable role in assessing optic pathway involvement and monitoring patients, the predictive value of preoperative OCT parameters is emphasized for estimating final visual acuity and visual field outcomes following neurosurgical treatment for CP. Importantly, both adult and pediatric cohorts show that preoperative RNFL and GCC thickness has prognostic value, as preserved RNFL is associated with a higher likelihood of postoperative visual recovery following surgical decompression [29]. OCT has emerged as a reliable tool for predicting visual outcomes in patients with CP by providing quantitative assessment of the RNFL and, in some studies, macular and GCC parameters. Multiple studies in adult and pediatric populations have consistently shown that preoperative RNFL thinning is associated with more severe visual field defects and reduced visual acuity [30,31,32,40]. Importantly, preoperative RNFL measurements have prognostic value: patients with relatively preserved RNFL are more likely to recover visual fields and acuity after surgical decompression [29,32,78]. Sectoral analysis of the RNFL enables the identification of characteristic patterns of fiber loss, with thickness measurements in specific sectors potentially offering greater predictive value for visual field deficits or, conversely, for the likelihood of visual field improvement [30,40]. Moon et al. [40] reported a significant correlation between preoperative RNFL thickness in the superior and temporal quadrants and postoperative visual field mean deviation. RNFL thickness in the temporal quadrant showed the strongest correlation with postoperative visual field outcomes in patients with CP. Garcia et al. [30] reported that RNFL thickness in the nasal sector was a good prognostic factor for improvement in the peripheral visual field. In adult patients with CP, Qiao et al. [78] found that greater inferior RNFL thickness was significantly associated with improved and preserved visual fields after surgery. The time to visual field improvement after neurosurgical intervention varied with the extent of preoperative RNFL damage [90]. In their analysis, Danesh et al. [90] reported the greatest visual field improvement in patients with thin RNFL between 6 weeks and 15 months after surgery, whereas in patients with thicker, normal RNFL, improvement occurred within up to 10 weeks postoperatively. Similarly, regarding visual acuity, Qiao et al. [78] reported that greater temporal RNFL thickness was associated with a higher likelihood of improvement and preservation of visual acuity after neurosurgical intervention. Thinner preoperative RNFL thickness was associated with worse visual acuity. In patients with normal preoperative RNFL, a significant improvement in mean visual acuity was observed after surgery, from 20/40 to 20/25, whereas no improvement was noted in patients with thin RNFL [68]. In the study by Danesh-Meyer et al. [90], at final follow-up, 97.5% of eyes with normal RNFL thickness achieved a visual acuity of 6/12 or better, compared with 88.2% of eyes with thin RNFL (*p* = 0.034). Furthermore, OCTA offers complementary information by quantifying microvascular parameters, including peripapillary and macular vessel density and choroidal perfusion, which have been shown to correlate with structural RNFL loss and postoperative visual outcomes in pediatric CP [60]. Zhang et al. [60] reported that patients with normal choroidal capillary density before surgery tended to show visual improvement. The baseline CCD cutoff value of approximately 38% was identified as a natural threshold for predicting visual prognosis after surgery [60]. Table 4 summarizes studies evaluating the predictive value of OCT- and OCTA-derived parameters for visual function and postoperative visual outcomes in patients with chiasmal compression and craniopharyngioma.

### 9.3. The Role of OCT in Postoperative Monitoring of Patients with CP

OCT has proven to be an essential tool for postoperative monitoring in patients with CP, providing an objective and quantitative assessment of retinal structures after surgical decompression. Both peripapillary RNFL and macular GCC measurements allow clinicians to track recovery or progression of optic pathway damage [32]. Mediero et al. [13] demonstrated that in pediatric patients, postoperative GCC and RNFL analysis correlated closely with visual field improvement and visual acuity recovery, highlighting the role of OCT in evaluating functional outcomes. Similarly, Meyer J. et al. [28] reported that pre- and postoperative RNFL thickness predicted visual recovery in patients undergoing surgery for parasellar tumors, supporting its prognostic utility. Macular analysis, particularly with SD-OCT, can detect subtle topographic changes in ganglion cell layers, including naso-temporal overlap, which may precede or exceed changes in peripapillary RNFL [73,77]. Recent evidence from Solari et al. [29] indicates that serial OCT measurements allow early identification of patients at risk of persistent visual deficits following endoscopic endonasal surgery for sellar and suprasellar lesions. Furthermore, Rakusiewicz-Krasnodębska et al. [64] evaluated RNFL thickness after transcranial craniotomy exclusively in a pediatric patient cohort. Tumor location, tumor volume, maximum tumor diameter, calcification, presence of a ventriculoperitoneal shunt, surgical technique, extent of resection, presence of Rosenthal fibers, and reoperation due to progression or recurrence were correlated with RNFL damage. Overall, OCT provides a noninvasive, reproducible method to monitor both focal and longitudinal structural changes, guiding postoperative management, risk stratification, and prognostication in patients with CP.

## 10. Limitations

Despite the growing evidence supporting the use of OCT in patients with CP, several limitations should be acknowledged. Most studies to date are retrospective and involve relatively small cohorts, which may limit the generalizability of the findings. Data on OCTA in this patient population remain scarce, limiting comprehensive evaluation of microvascular changes associated with optic pathway compression. Standardized protocols for image acquisition, segmentation, and interpretation are also lacking, contributing to potential variability between centers and studies. In pediatric patients, cooperation can be challenging, often resulting in suboptimal image quality. Moreover, in patients with low visual acuity, the peripheral fixation required for reliable RNFL assessment is frequently not possible, further limiting the applicability of RNFL measurements in routine practice. These limitations underscore the need for prospective, multicenter studies and standardized guidelines to optimize the clinical utility of OCT and OCTA in both preoperative and postoperative evaluation of patients with CP.

## 11. Future Directions

OCT provides a practical, noninvasive tool for both diagnosing and postoperatively monitoring patients with CP, enabling clinicians to assess structural damage, track disease progression, and inform surgical planning. Based on our experience, Table 5 summarizes recommended OCT assessments, including key parameters and suggested follow-up intervals, to guide routine clinical practice. Importantly, OCT should not be used as a standalone tool, its findings must be interpreted in conjunction with visual acuity, visual field testing, and fundoscopic examination and electrophysiology to provide a comprehensive assessment of optic pathway function. The proposed monitoring schedule is designed to detect early visual deterioration related to tumor progression or surgical intervention. BCVA assessment is the primary functional test in cooperative children, whereas VEP provides an objective evaluation of the visual pathway in patients with severe visual impairment (BCVA < 0.1) or in preverbal children. The selection of additional tests, such as visual field examination, depends on the child’s age and ability to cooperate. Future directions include the integration of OCTA to evaluate retinal microvascular changes, the development of standardized imaging and analysis protocols, and prospective multicenter studies to validate OCT-based biomarkers as predictors of visual outcomes. Such advances may further refine risk stratification, optimize postoperative monitoring, and improve individualized patient care.

## 12. Conclusions

OCT and OCTA represent powerful, noninvasive tools for evaluating retinal and optic nerve integrity in patients with CP. Preoperative and postoperative OCT assessments provide prognostic information, guide surgical management, and support long-term monitoring of visual function. Integration of OCT into routine clinical practice enhances early detection of optic pathway damage and improves patient outcomes.

## Figures and Tables

**Table 1:** Clinical studies evaluating peripapillary RNFL thickness using OCT in patients with craniopharyngioma.

Author (Year)	Study Population	OCT Modality	Assessed Parameters	Key Findings Related to RNFL
Danesh-Meyer et al. [32] (2006)	Children and adults with chiasmal compression (2 CP)	TD-/SD-OCT	RNFL	Patients with chiasmal compression had statistically significantly lower RNFL thickness than the control group.
Danesh-Meyer et al. [68] (2008)	Adults with chiasmal compression (1 CP)	OCT	RNFL	Preoperative RNFL thickness predicts postoperative visual recovery (visual acuity and visual field).
Moon et al. [40] (2011)	Adults with chiasmal compression (2 CP)	SD-OCT	RNFL	Preoperative RNFL provides prognostic information for visual outcome.
Moon et al. [72] (2012)	Adults with chiasmal compression (7 CP)	SD-OCT	RNFL	RNFL measurements are effective in detecting chiasmal compression.
Bialer et al. [31] (2013)	Children with CP	SD-OCT	RNFL	Patients with CP had statistically significantly lower RNFL thickness than the control group.
Akashi et al. [73] (2014)	Adults with chiasmal compression neuropathy (5 CP)	SD-OCT	RNFL, macular analysis	Patients with chiasmal compression had statistically significantly lower RNFL thickness than the control group.
Garcia et al. [30] (2014)	Adults with optic chiasm compression (5 CP)	SD-OCT	RNFL	RNFL thickness predicts postoperative peripheral visual field recovery.
Mediero et al. [13] (2015)	Children with CP	SD-OCT	RNFL	RNFL correlates with visual acuity and visual field defects.
Yang et al. [77] (2016)	Primary CP (Children and adult)	FD-OCT	RNFL	Significant RNFL thinning compared with controls before treatment.
Gil-Simoes et al. [75] (2019)	Intrachiasmatic CP—case report	SD-OCT	RNFL	RNFL useful for assessing visual outcome after complete tumor resection.
Ju et al. [67] (2019)	Adults with chiasmal compression (8 CP)	SD-OCT	RNFL	Patients with optic tract edema demonstrated greater peripapillary RNFL thinning and worse visual outcomes.
Lee et al. [14] (2021)	Children with CP	SD-OCT	RNFL	Patients with CP had statistically significantly lower postoperative RNFL thickness than the control group.
Qiao et al. [78] (2022)	Adults with CP	SD-OCT	RNFL	Preoperative peripapillary RNFL thickness were significant predictors of postoperative visual outcomes.
Rakusiewicz-Krasnodębska et al. [64] (2025)	Children with CP	SD-OCT	RNFL	Significant RNFL thinning associated with optic nerve compression; RNFL thickness correlated with severity of visual impairment and reflected structural damage of the anterior visual pathway.
Shinohara et al. [66] (2025)	Adults with CP	SD-OCT	RNFL	Patients with adult CP demonstrated significant thinning of peripapillary RNFL

CP: craniopharyngioma; RNFL: retinal nerve fiber layer; OCT: optical coherence tomography; TD-OCT: time-domain optical coherence tomography; SD-OCT: spectral-domain optical coherence tomography; FD-OCT: Fourier-domain optical coherence tomography; VA: visual acuity; VF: visual field.

**Table 2:** Clinical studies evaluating ganglion cell complex using OCT in patients with CP and chiasmal compression.

Author (Year)	Study Population	OCT Modality	Assessed Parameters	Key Findings Related to GCC
Moon et al. [40] (2011)	Adults with chiasmal compression (2 CP)	SD-OCT	GCC	GCC provide prognostic value for visual function.
Akashi et al. [73] (2014)	Adults with chiasmal compression neuropathy (5 CP)	SD-OCT	GCC, Macular analysis	Patients with chiasmal compression had statistically significantly lower GCC thickness than the control group.
Mediero et al. [13] (2015)	Children with CP	SD-OCT	GCC	GCC thinning correlates with visual acuity and visual field loss.
Yang et al. [77] (2016)	Primary CP	FD-OCT	GCC	Significant GCC thinning indicating neuronal loss.
Gil-Simoes et al. [75] (2019)	Intrachiasmatic CP-case report	SD-OCT	GCC	GCC helpful in postoperative assessment of visual outcome.
Lee et al. [14] (2021)	Children with CP	SD-OCT	GCL	Patients with CP had statistically significantly lower postoperative GCL thickness than the control group.

Summary of studies evaluating ganglion cell complex (GCC) and ganglion cell layer (GCL) thickness using optical coherence tomography (OCT in patients with craniopharyngioma (CP) or chiasmal compression, highlighting their diagnostic and prognostic relevance for visual function. CP: craniopharyngioma; OCT: optical coherence tomography; SD-OCT: spectral-domain optical coherence tomography; FD-OCT: Fourier-domain optical coherence tomography; GCC: ganglion cell complex; GCL: ganglion cell layer; VA: visual acuity; VF: visual field.

**Table 3:** Clinical studies evaluating retinal vessel density using OCTA in patients with CP and chiasmal compression.

Author (Year)	Study Population	OCTA Region	Assessed OCTA Parameters	Key Findings and Clinical Implications
Higashiyama et al. [34] (2016)	Adults with chiasmal compression (1 CP)	Peripapillary	retinal and peripapillary vessel density	Patients with chiasmal compression demonstrated reduced retinal and peripapillary perfusion on OCTA.
Suzuki et al. [41] (2020)	Adults with chiasmal compression (2 CP)	Peripapillary and macular	Vessel density (superficial retinal plexus), circumpapillary perfusion	Reduced peripapillary and macular vessel density correlated with RNFL/GCC thinning and visual field loss
Lee et al. [14] (2021)	Adults with chiasmal compression due to pituitary tumors (3 CP)	Parafoveal and peripapillary	Vessel density (superficial and deep retinal plexus), circumpapillary perfusion	Decompression surgery resulted in partial recovery of parafoveal and peripapillary vessel density. Improvements in OCTA metrics correlated with structural recovery of RNFL/GCC and functional visual outcomes.
Wang et al. [83] (2021)	Adults with chiasmal compression (3 CP)	Peripapillary	Vessel density (superficial and deep retinal plexus), radial peripapillary capillary density	Patients exhibited significant reduction in peripapillary vessel density associated with RNFL thinning and visual field deficits.
Lee et al. [89] (2020)	Adults with chiasmal compression (3 CP)	Parafoveal and peripapillary	Vessel density (superficial retinal plexus), circumpapillary perfusion	Reduced parafoveal and peripapillary vessel density were significant predictors of postoperative visual field recovery.
Zhang et al. [60] (2022)	Children with CP	Macular and choroidal layers	Choroidal capillary density, macular vessel density	OCTA-derived choroidal perfusion parameters predicted postoperative visual outcomes.
Lee et al. [42] (2022)	Children with CP	Parafoveal and peripapillary	Vessel density (superficial plexus), FAZ-related metrics	Significant reduction in parafoveal and peripapillary vessel density associated with visual dysfunction.
Ergen et al. [88] (2023)	Adults with sellar/parasellar tumors (4 CP)	Peripapillary and macular	Vessel density (superficial and deep retinal plexus), circumpapillary perfusion, FAZ metrics	Endoscopic decompression led to partial recovery of peripapillary and macular vessel density, which correlated with structural RNFL and GCC improvements and visual function recovery.

Summary of optical coherence tomography angiography (OCTA) studies evaluating retinal, peripapillary, macular, and choroidal microvascular alterations in patients with craniopharyngioma (CP) or chiasmal compression, and their associations with structural damage and visual function. CP: craniopharyngioma; OCTA: optical coherence tomography angiography; RNFL: retinal nerve fiber layer; GCC: ganglion cell complex.

**Table 4:** Predictive value of OCT- and OCTA-derived parameters for visual function and postoperative visual outcomes in patients with chiasmal compression and craniopharyngioma.

Author (Year)	Study Population	OCT Modality/Parameters	Visual Function Assessed	Key Findings/Predictive Value
Danesh-Meyer et al. [32] 2006	Adults and children with chiasmal compression (2 CP)	RNFL	Visual field (VF) sensitivity	RNFL thickness correlates strongly with VF sensitivity; thinning predicts severity of visual deficits.
Moon et al. [40] 2011	Adults with chiasmal compression (2 craniopharyngiomas)	RNFL, photopic negative response	VF, visual acuity (VA)	Preoperative RNFL and functional measurements predict postoperative visual recovery.
Garcia et al. [30] 2014	Adults with optic chiasm compression	RNFL	VF	RNFL thickness predicts postoperative peripheral VF recovery; sectoral analysis identifies pattern of fiber loss. Nasal retinal nerve fiber layer (RNFL) thickness was a good prognostic factor for peripheral visual field recovery.
Danesh-Meyer et al. [68] 2008	Adults with parachiasmal tumors	RNFL	VF, VA	Preoperative RNFL thickness predicts the likelihood of postoperative visual recovery (visual acuity and visual field).
Moon et al. [72] 2012	Adults with chiasmal compression	RNFL	VF	Sectoral RNFL analysis improves the detection of asymmetric or subtle visual deficits.
Solari et al. [29] 2022	Adults with sellar-suprasellar lesions	SD-OCT RNFL	VF, VA	Serial RNFL measurements allow early prediction of visual recovery after endoscopic surgery.
Qiao et al. [78] 2022	Adults with craniopharyngioma	RNFL, GCC	VF, VA	Preserved RNFL and GCC predict favorable postoperative visual outcomes.
Danesh-Meyer et al. [90] 2015	Adults with pituitary tumors	RNFL	VF	RNFL thickness predicts postoperative visual recovery; an objective biomarker for surgical planning.
Zhang et al. [60] 2022	Pediatric CP	OCTA: peripapillary & macular vessel density, choroidal capillaries	VF, VA	Microvascular parameters correlate with RNFL loss and predict postoperative visual outcomes.
Santorini et al. [18] (2022)	Patients with chiasmal compression	SD-OCT	RNFL, GCC, GCL	RNFL and macular parameters provide complementary information.

Overview of studies evaluating structural and microvascular biomarkers derived from optical coherence tomography (OCT) and optical coherence tomography angiography (OCTA) in patients with chiasmal compression or craniopharyngioma (CP), with a focus on their associations with visual function and their predictive value for postoperative visual recovery. CP: craniopharyngioma; OCT: optical coherence tomography; OCTA: optical coherence tomography angiography; SD-OCT: spectral-domain optical coherence tomography; RNFL: retinal nerve fiber layer; GCC: ganglion cell complex; GCL: ganglion cell layer; VF: visual field; VA: visual acuity.

**Table 5:** Ophthalmological Assessment and Follow-Up Schedule in Pediatric Patients with CP.

At the Time of Diagnosis:
Evaluation of distance and near best-corrected visual acuity (BCVA) * [* VEP (visual evoked potentials) is recommended for patients with visual acuity < 0.1, and for other patients when feasible and indicated. VEP assessment is recommended in preverbal children.]
2. Assessment of ocular motility, ocular alignment, anterior segment, and fundus examination.
3. Visual field testing is advised for children over 8 years who can cooperate, with attempts made in younger children when possible.
4. Optical coherence tomography (OCT) with evaluation of the retinal nerve fiber layer (RNFL) and ganglion cell complex (GCC)
Frequency of examinations:
At the time of diagnosis
2. Preoperatively—performed only when the interval between diagnosis and surgery exceeds three days.
3. 7–10 days post-surgery, or sooner in case of clinical indications or concerning symptoms.
4. 3 months after surgery, earlier if clinical indications or concerning symptoms occur
5. Subsequently, every 6 months, earlier if clinical indications or concerning symptoms occur

BCVA, best-corrected visual acuity; VEP, visual evoked potentials; OCT, optical coherence tomography; RNFL, retinal nerve fiber layer; GCC, ganglion cell complex.

## Data Availability

The data presented in this study are available on request from the corresponding author. The data are not publicly available due to ethical restrictions and the need to protect patient confidentiality.
